# DNA double-strand break repair: a theoretical framework and its application

**DOI:** 10.1098/rsif.2015.0679

**Published:** 2016-01

**Authors:** Philip J. Murray, Bart Cornelissen, Katherine A. Vallis, S. Jon Chapman

**Affiliations:** 1 Division of Mathematics, University of Dundee, Dundee, UK; 2 Department of Oncology, University of Oxford, Oxford, UK; 3 Department of Mathematics, University of Oxford, Oxford, UK

**Keywords:** radiobiology, mathematical model, DNA damage repair

## Abstract

DNA double-strand breaks (DSBs) are formed as a result of genotoxic insults, such as exogenous ionizing radiation, and are among the most serious types of DNA damage. One of the earliest molecular responses following DSB formation is the phosphorylation of the histone H2AX, giving rise to *γ*H2AX. Many copies of *γ*H2AX are generated at DSBs and can be detected *in vitro* as foci using well-established immuno-histochemical methods. It has previously been shown that anti-*γ*H2AX antibodies, modified by the addition of the cell-penetrating peptide TAT and a fluorescent or radionuclide label, can be used to visualize and quantify DSBs *in vivo*. Moreover, when labelled with a high amount of the short-range, Auger electron-emitting radioisotope, ^111^In, the amount of DNA damage within a cell can be increased, leading to cell death. In this report, we develop a mathematical model that describes how molecular processes at individual sites of DNA damage give rise to quantifiable foci. Equations that describe stochastic mean behaviours at individual DSB sites are derived and parametrized using population-scale, time-series measurements from two different cancer cell lines. The model is used to examine two case studies in which the introduction of an antibody (anti-*γ*H2AX-TAT) that targets a key component in the DSB repair pathway influences system behaviour. We investigate: (i) how the interaction between anti-*γ*H2AX-TAT and *γ*H2AX effects the kinetics of H2AX phosphorylation and DSB repair and (ii) model behaviour when the anti-*γ*H2AX antibody is labelled with Auger electron-emitting ^111^In and can thus instigate additional DNA damage. This work supports the conclusion that DSB kinetics are largely unaffected by the introduction of the anti-*γ*H2AX antibody, a result that has been validated experimentally, and hence the hypothesis that the use of anti-*γ*H2AX antibody to quantify DSBs does not violate the image tracer principle. Moreover, it provides a novel model of DNA damage accumulation in the presence of Auger electron-emitting ^111^In that is supported qualitatively by the available experimental data.

## 1. Introduction

DNA double-strand breaks (DSBs), one of the most lethal types of DNA damage, can be caused by factors such as oncogenic stress, genomic instability, several anti-cancer treatments and ionizing radiation including radiation therapy (IR). Moreover, *in vitro* analyses have shown that the ability of various treatments to cause DSBs is directly related to treatment efficacy [1,2]. Therefore, the ability to measure the extent of DSB damage in tumour tissue could provide a prognostic biomarker during cancer therapy.

Although DSBs cannot be measured directly, several assays that provide a secondary marker of the extent of DNA damage can be used to visualize and quantify the cell's response to DSB damage and the signalling pathways of DNA damage response (DDR). One of the earliest and universal events during DDR is the phosphorylation, by the kinases ATM, ATR and DNK-PKcs, of the histone isoform H2AX on serine residue 139 (P-S139) to form *γ*H2AX [3]. *γ*H2AX forms foci of up to a few thousand copies around sites of DSB, and *γ*H2AX foci are widely used to monitor DSB repair *in vitro* and *ex vivo* (for reviews, see [4–9]). The phosphorylation event is essential, as in its absence DDR occurs significantly slower [10,11]. P-S139-H2AX acts as a scaffold for the recruitment of other DNA damage repair proteins, including the MRN complex, MDC1, ATM and BRCA1 [4].

Previously, Cornelissen *et al*. [12] developed a method for imaging DSBs *in vivo* in which anti-*γ*H2AX antibodies were conjugated to the cell-penetrating peptide TAT, to allow cellular internalization, and to radionuclides or fluorophores, to allow SPECT and fluorescence microscopy, respectively. Here, we present a framework that describes dynamic behaviour in this system and allows us to study perturbations.

Previous mathematical models of DSB repair mechanisms (e.g. [13–15]) have described the sequential construction of complexes that are essential for DSB repair. Typically, systems of ordinary differential equations are used to describe concentrations of relevant complexes. However, as there is not currently a robust quantification of molecular behaviours at individual foci, these models are typically over-parametrized. Moreover, when they are parametrized, the link between available experimental data, made by counting the numbers of DSBs and *γ*H2AX foci across populations of cells, and underlying molecular networks is not formalized. In another body of work, Foray and co-workers (e.g. [16]) develop models that describe the phenomenology of foci appearance and disappearance. These models attempt to describe observations without explicitly accounting for molecular details. As the models have relatively few parameters, they offer a framework for robustly quantifying foci kinetics.

In this paper, we develop a framework in which the simulation of underlying molecular processes can be formally related to experimental observations. The resulting differential equation models differ from previous works in that explicit assumptions made at the molecular scale emerge in the resulting population-scale equations. The model is parametrized using available data from two cancer cell lines and two case studies are considered in which the model is used to study experimentally motivated perturbations in which cell populations are treated with an anti-*γ*H2AX antibody.

## 2. Methods

### 2.1. Experimental methods

MCF7 and MDA-MB-468 human breast cancer cells (LGC Standards, Teddington, Middlesex, UK) were cultured as previously described [12]. Cells were tested and authenticated by the provider and their cumulative time in culture was less than six months following retrieval. Rabbit polyclonal anti-*γ*H2AX antibodies (Calbiochem), or non-specific rabbit IgGs were conjugated to TAT-peptide (GRKKRRQRRRPPQGYG; Cambridge peptides, Cambridge, UK), to produce anti-*γ*H2AX-TAT and rabbit IgG-TAT (rIgG-TAT), as previously described [12,17]. The bispecific metal ion chelator, pSCN-BnDTPA, was conjugated to antibody-TAT, to allow radiolabelling with varying amounts of ^111^In to produce ^111^In-anti-*γ*H2AX-TAT or ^111^In-rIgG-TAT of a range of specific activities (the amount of ^111^In per gram of antibody), as previously described [12,17].

To determine the influence of anti-*γ*H2AX-TAT on *γ*H2AX foci kinetics after irradiation, cells were grown in 96-well plates and exposed to ^111^In-labelled (1–4 MBq µg^−1^) or non-labelled (0 MBq µg^−1^) anti-*γ*H2AX-TAT, rIgG-TAT (0–0.5 mg ml^−1^) or a molar equivalent of TAT-peptide (0–0.06 mg ml^−1^). After incubation at 37°C for 1 h, cells were irradiated (4 Gy) using a ^137^Cs irradiator (1.0 Gy min^−1^; Gulmay). To avoid DDR signalling pathway activation during irradiation, cells were irradiated on ice. At selected times, cells were washed, fixed and stained for *γ*H2AX using mouse anti-*γ*H2AX antibodies (Millipore; 1 : 1500; 1 h, 37°C) and Alexa fluor 488-labelled goat anti-mouse antibodies (Invitrogen; 1 : 250; 1 h, 37°C) as previously described [12]. Nuclei were counterstained with DAPI. Images were acquired using an IN Cell Analyser (GE Healthcare) and the number of *γ*H2AX foci per cell was determined using proprietary IN Cell Analyser analysis software.

To measure the influence of anti-*γ*H2AX-TAT on the extent of DNA DSB damage, cell suspensions (5 × 10^5^ cells in 500 µl of cell medium) were exposed to anti-*γ*H2AX-TAT or rIgG-TAT (0.5 µg ml^−1^). After incubation for 1 h at 37°C, cells were irradiated on ice (4 Gy) or sham-irradiated. After incubation at 37°C, neutral comet assays (NCA) were performed at selected time points, using the Trevigen COMETS kit (Trevigen, Helgerman, CT, USA), according to the manufacturer's guidelines. The Olive tail moment (OTM), a measure of the number of DNA DSBs, was determined using software developed in-house, as previously described [17].

To measure the influence of ^111^In-anti-*γ*H2AX-TAT on clonogenic survival, cell suspensions (2 × 10^5^ cells in 200 µl of medium) were incubated with ^111^In-anti-*γ*H2AX-Tat or ^111^In-rIgG-TAT (0.05 µg ml^−1^, specific activities 0–4 MBq µg^−1^) for 1 h at 37°C to allow internalization and nuclear accumulation of radioimmunoconjugates (RICs). Cells were exposed to *γ*-radiation (0 or 10 Gy) and incubated for 24 h at 37°C. An aliquot of cells was plated in DMEM with 10% fetal bovine serum (FBS) (20% for MDA-MB-468 cells) and incubated at 37°C and 5% CO_2_. Colonies were counted after one to two weeks and the surviving fraction calculated, as previously described [17].

### 2.2. Model development

Although there are multiple molecular components (e.g. ATM, ATR, H2AX, BRCA1, the MRN complex, MDC1, DNA-PKcs) and processes (e.g. diffusion, binding, phosphorylation) involved in the repair of a DSB, in this study our approach is to develop a theoretical framework that describes fundamental processes that can be constrained by currently available data.

We let the variable *X*(*t*) represent a telegraph-like signal that describes whether or not there is a DSB at a particular site such that when a DSB is present, the telegraph signal is on (*X* = 1) and repair processes can occur. Conversely, when the telegraph signal is off (*X* = 0), recruitment of repair signalling molecules does not occur. Crucially, the switch from the on to off states is coupled to the dynamics of repair processes at a given site. The second dependent variable, *Z*(*t*), represents the number of phosphorylated H2AX molecules at a given site. It is chosen as *γ*H2AX is known to play a crucial role in DSB repair and *γ*H2AX foci are a measurable quantity.

In contrast to histones, which are fixed in a reference frame with DNA, numerous molecules that diffuse in the local environment accumulate at DSB sites to initiate and advance repair (e.g. pATM, ATR, DNA-PKcs). As time-series quantification for each of these variables are not readily available, they are grouped together in the variable *Y*(*t*) which denotes the number of bound, activated diffusible molecules at a given DSB site (e.g. pATM). We assume that the presence of bound and activated diffusible molecules is necessary for DSB repair and that the accumulation of such molecules is part of a positive feedback loop with H2AX such that Y both causes the phosphorylation of H2AX (forming *γ*H2AX) and is upregulated by phosphorylated H2AX. Additionally, we assume that the unphosphorylated H2AX is in abundance, hence its concentration is approximately constant. As phosphorylation is many times faster than recruitment, the recruitment and (auto-)phosphorylation of these species is treated as one single step. We note that for brevity below, the variable *Y*(*t*) is referred to as pATM but stress that it could represent any diffusible species that binds at DSB site and is necessary for DNA repair.

**Figure 1.**
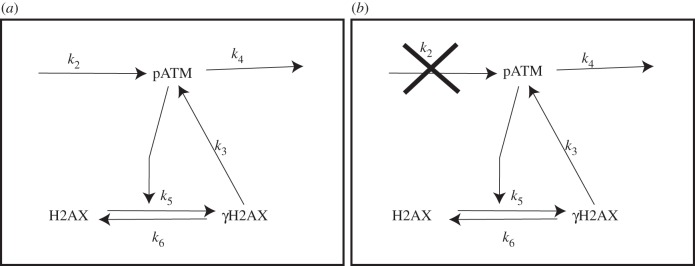
A schematic of the *γ*H2AX–pATM interaction network. In the presence of a DSB (*a*), pATM molecules are recruited to the DSB site at rate *k*_2_, dissociate at rate *k*_4_ and are further recruited by *γ*H2AX at rate *k*_3_. H2AX gets phosphorylated and *γ*H2AX dephosphorylated at rates *k*_5_ and *k*_6_, respectively. In the absence of a DSB (*b*), a stable steady-state exists in which the concentrations of pATM and *γ*H2AX are zero.

The interactions described above are formalized as follows (see figure 1 for a schematic illustration). In the time interval [*t*, *t* + Δ*t*]: (i) DSB repair is dependent on the number of recruited pATM molecules such that the probability of a repair occurring in time Δ*t* is *k*_1_*Y*(*t*)Δ*t*; (ii) pATM molecules are recruited to a DSB site with probability *k*_2_*X*(*t*)Δ*t* such that in the presence of a DSB (*X* = 1), recruitment occurs at rate *k*_2_ and upon repair (*X* = 0), recruitment stops; (iii) pATM molecules are recruited by phosphorylated H2AX with probability *k*_3_*Z*(*t*)Δ*t*; (iv) H2AX gets phosphorylated to *γ*H2AX with probability *k*_5_*Y*(*t*)Δ*t* and (v) dissociation of pATM from the DSB site and dephosphorylation of *γ*H2AX occur with probabilities *k*_4_*Y*(*t*)Δ*t* and *k*_6_*Z*(*t*)Δ*t*, respectively.

Defining *P*(*X*, *Y*, *Z*; *t*) to be the probability that at time *t*, a DNA site is in state *X*, with *Y* molecules of bound pATM and *Z* molecules of phosphorylated H2AX, the stochastic processes outlined in the previous paragraph are described by the master equation
2.1
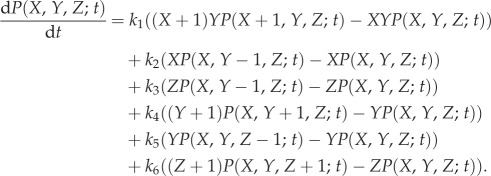
Note that we use the convention that *P*(2, *Y*, *Z*; *t*) = 0.

**Figure 2.**
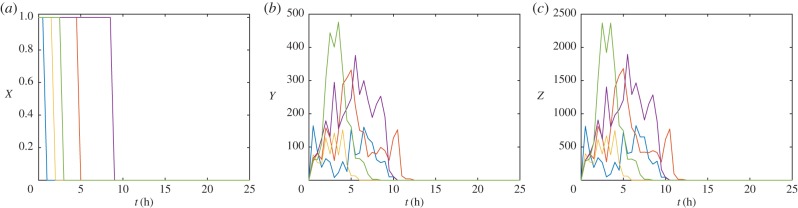
Sample realizations of equation (2.1). Lines depict five different stochastic simulations. (*a*) The presence of a DSB, *X*(*t*) is plotted against time, *t*. (*b*) The number of pATM molecules, *Y*(*t*), is plotted against time, *t*. (*c*) The number of *γ*H2AX molecules, *Z*(*t*), is plotted against time, *t*. Parameter values as in table 1. (Online version in colour.)

Using Gillespie's stochastic simulation algorithm (SSA), solutions of equation (2.1) for initial conditions in which there is a DSB at a given site and zero molecules of pATM and *γ*H2AX were calculated (figure 2). In a typical simulation, the diffusible molecules bind at the DSB site, leading to the accumulation of *γ*H2AX and further accumulation of pATM. Eventually, as a consequence of the presence of diffusible molecules, the telegraph signal is switched off. Consequently, dissociation and dephosphorylation of repair molecules become the dominant processes and the system eventually reaches a steady state where the telegraph signal is off and there are no longer any bound repair molecules.

**Table 1.** Numerical values for fitted rate parameters. Solutions of equations (2.8) were calculated and the parameter set {*k*_1_, *k*_2_, … , *k*_6_,} that minimizes equation (3.3) was determined. All rate constants have unit h^−1^.

parameter	MDA-MB-468	MCF7
*k*_1_	0.0032	0.02
*k*_2_	159	1236
*k*_3_	14	220
*k*_4_	71	687
*k*_5_	1056	1765
*k*_6_	211	565

Given that experiments are typically performed over thousands of DSBs (approx. 40 DSBs per cell per Gy [18]), we define the stochastic means
2.2
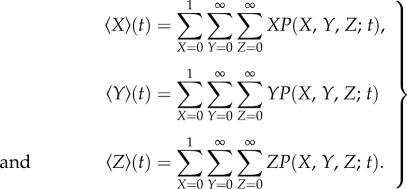
Upon differentiation of the above quantities with respect to time, we obtain, using equation (2.1) and some standard manipulations,
2.3
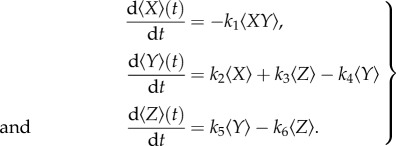


### 2.3. Moment closure

The first of equations (2.3) contains a nonlinear term that requires a further approximation to define a closed model.

#### 2.3.1. An *ad hoc* closure

The simplest closure is to assume that
2.4
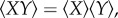
which would be the case if 〈*X*〉 and 〈*Y*〉 were independent (uncorrelated). Equations (2.3) then take the form
2.5
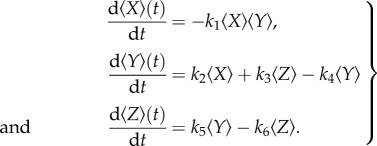


#### 2.3.2. Conditional means

We can perform a higher-order closure by introducing conditional means. We have
2.6
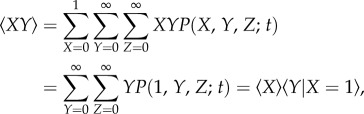
where 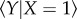 is the conditional mean value of the variable *Y* when *X* = 1. Defining governing equations for the conditional means yields


2.7
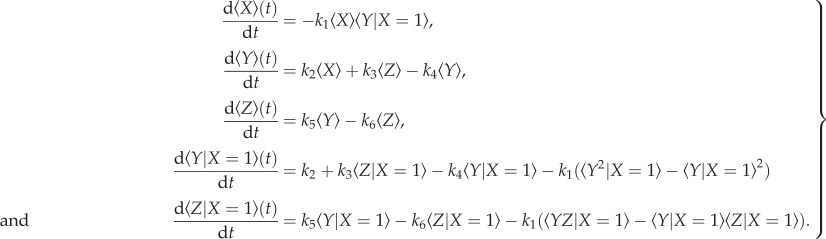


Closing the model by assuming that the conditional (co)variances are negligible, we obtain
2.8
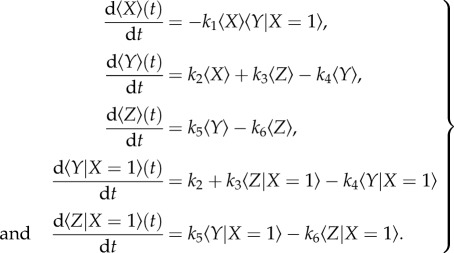


## 3. Results

### 3.1. Examining stochastic model behaviour

**Figure 3.**
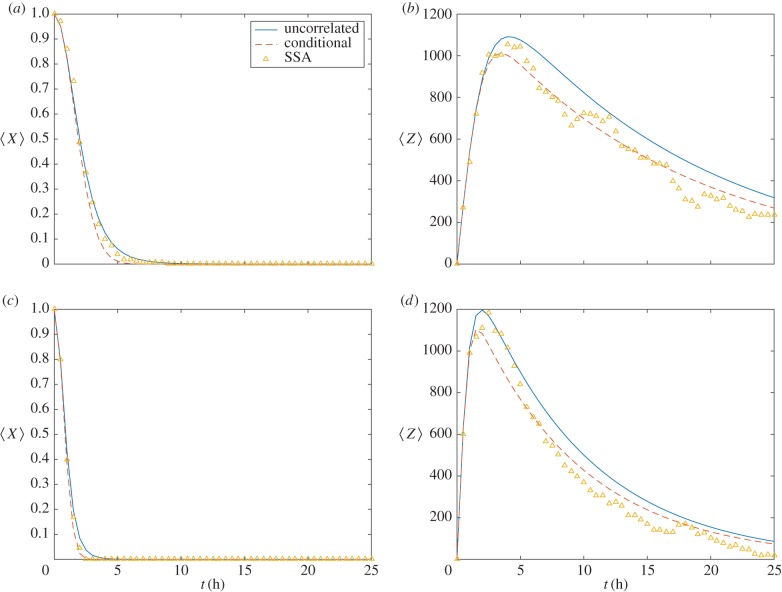
Investigating the moment closure approximation used to derive equations (2.5). (*a*,*c*) Expected number of DSBs, 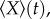 is plotted against time, *t*. (*b*,*d*) Expected number of *γ*H2AX molecules, 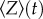, is plotted against time, *t*. Realizations of equation (2.1) are averaged (markers) and compared with solutions of equations (2.8) (dashed lines) and (2.5) (solid lines). Parameter values defined in table 1. MDA-MB-468 (*a*,*b*); MCF7 (*c*,*d*). (Online version in colour.)

To ensure that the closures presented in §2.3 provide a sufficiently accurate description of the mean behaviours of the solutions presented in figure 2, sample means, calculated from averaging over 1000 stochastic realizations, are compared with solutions of the differential equation model (figure 3). These numerical results illustrate that, at least for the parameter values chosen, the differential equation model is an accurate representation of the underlying stochastic model.

### 3.2. Parameter identification

Defining *χ*_1_(*t*) and *χ*_2_(*t*) to be the time-series measurements for the average numbers of DSBs and *γ*H2AX foci per cell, respectively (e.g. see §2.1 for further details), we seek the optimal parameter set {*k*_1_, *k*_2_, …,*k*_6_} that describes the observations for a given cell line.

The variable 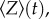 which represents the expected number of *γ*H2AX molecules at a given focus, is related to the experimentally measured quantity *χ*_2_(*t*), the number of observable *γ*H2AX foci, by assuming that
3.1
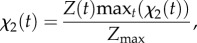
thus ensuring that the model solution recapitulates the number of *γ*H2AX molecules thought to be at a typical focus. Notably, previous authors have made similar assumptions to fit molecular models to foci kinetic data. In §3.1, we will use the SSA to check the validity of this assumption *a posteriori*.

The observation that DSB repair occurs significantly slower (approx. 10 times) in the absence of H2AX [10,11] is captured by defining, in the absence of explicit time-series measurements,
3.2
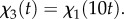


To represent the case of no *γ*H2AX, this quantity is fitted to the model by solving equations (2.5) with the parameter *k*_5_ = 0. We denote such solutions using a barred notation (i.e. the number of DSBs in a model solution representing the case of no *γ*H2AX is given by 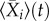).

Combining the above assumptions, the least-squares error, given by


**Figure 4.**
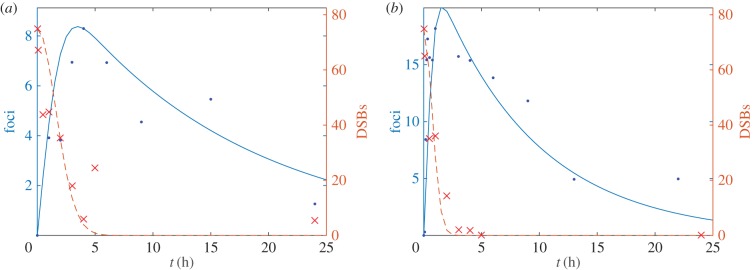
*γ*H2AX foci (solid lines, dots) and DSB (dashed lines, crosses) number are plotted against time for (*a*) MDA-MB-468 and (*b*) MCF7 cells. Experimental data are denoted by markers. The solution of the averaged model (2.8) (lines) was computed using the optimized parameter sets presented in table 1. (Online version in colour.)

3.3
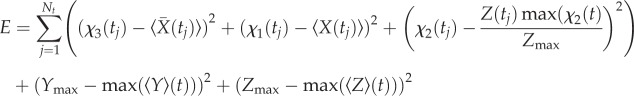


is minimized using the Nelder–Mead simplex direct search method implemented via Matlab's *fminsearch* function. In table 1 and figure 4, the parameter values fitted to the MCF7 and MDA-MB-468 cell lines are presented. We note that the values for the constants presented in table 2 are estimated counts of molecules at individual foci.

**Table 2.** *A priori* assumed quantities used in the fitting of the rate constants defined in table 1.

parameter	value	description
*Y*_max_	300	maximum number of bound pATM molecules per DSB
*Z*_max_	1000	number of *γ*H2AX molecules in focus
Z*	200	number of *γ*H2AX molecules needed to make focus detectable

### 3.3. Number measured foci is proportional to mean number of *γ*H2AX molecules

**Figure 5.**
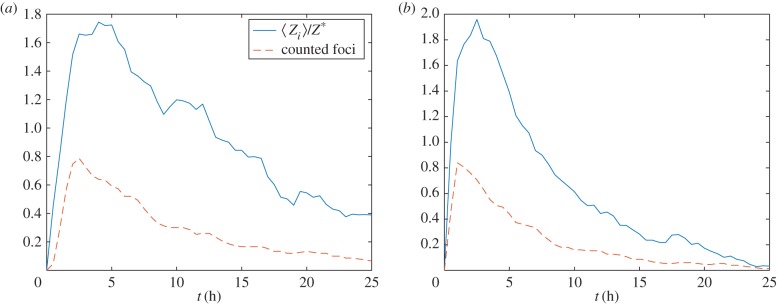
The average number of detectable *γ*H2AX foci (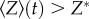, solid line) and *γ*H2AX molecules (〈*Z*〉(*t*), dashed line) are plotted against time. (*a*) MDA-MB-468 and (*b*) and MCF7. Realizations of equation (2.1) were calculated using Gillespie's algorithm. Parameter values as in table 1. (Online version in colour.)

Both in the parametrization described in §3.2 and in previous studies, it has been assumed that the experimentally measured number of observable foci is proportional to the total number of *γ*H2AX molecules counted across a number of *N*_DSB_ foci and averaged over an ensemble of realizations [13]. The stochastic model is used to investigate this assumption as follows: in a given stochastic realization, we determine that a *γ*H2AX focus is detectable under the microscope if the number of *γ*H2AX molecules exceeds some threshold, Z*, and calculate the expected number of visible foci in a population of *N*_DSB_ DSBs over an ensemble of realizations. In figure 5, we show, that for the parameter values chosen, the counted number of foci is proportional to the mean number of *γ*H2AX molecules.

## 4. Case study

In this case study, we explore how the proposed framework can be used to understand modulation of the DSB repair system by exogenous agents. In each of the subsections below, we present an experimentally motivated problem, apply the model developed above, and interpret the biological implications of the results.

### 4.1. Influence of *γ*H2AX-TAT

#### 4.1.1. Model extension and application

**Figure 6.**
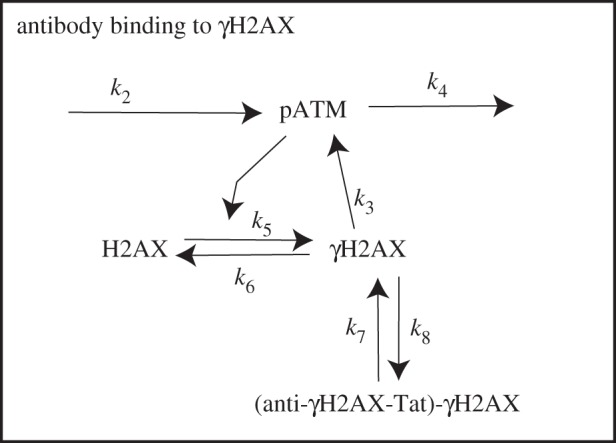
A schematic of inclusion of the anti-*γ*H2AX antibody.

To account for the effect of the anti-*γ*H2AX-TAT antibody, it is assumed that anti-*γ*H2AX-TAT binds reversibly to *γ*H2AX and that the bound complex is inert (i.e. it prevents interaction of *γ*H2AX with pATM, see schematic diagram presented in figure 6). Following a similar procedure to that outlined in §2.2 (see appendix A), we obtain
4.1
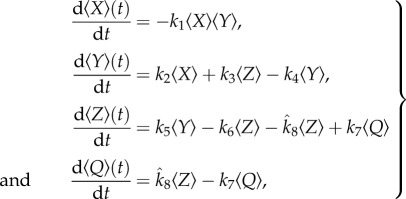
where 〈*Q*〉(*t*) is the expected numbers of bound antibody–*γ*H2AX molecules,
4.2
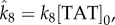
[TAT]_0_ is the concentration of anti-*γ*H2AX-TAT antibody, and *k*_7_ and *k*_8_ are dissociation and binding rates, respectively.

**Figure 7.**
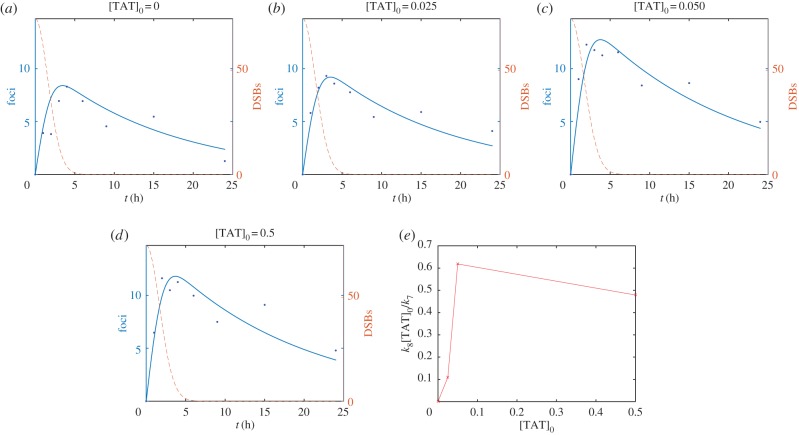
*γ*H2AX foci (solid lines, dots) and DSB (dashed lines) number are plotted against time. (*a*) [TAT]_0_ = 0, (*b*) [TAT]_0_ = 0.025, (*c*) [TAT]_0_ = 0.05 and (*d*) [TAT]_0_ = 0.5 µg ml^−1^. Lines represent solution to equations (4.1). Markers represent experimental observations from MDA-MB-468 cell line. (*e*) Fitted value of the parameter grouping *k*_8_[TAT]_0_/*k*_7_ is plotted against [TAT]_0_. (Online version in colour.)

Given the fitted values for parameters *k*_1_, *k*_2_, … , *k*_6_ defined in table 1, a prediction of the model is that the parameter combination 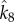 should increase linearly with the amount of anti-*γ*H2AX-TAT ([TAT]_0_) added to cells. In figure 7, this prediction is tested by fitting the parameter 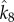 to foci data measured at different antibody concentrations. Notably, at low antibody concentration the model prediction is observed but at the high antibody concentration there is a saturation effect that is not predicted by the model. A further prediction of the model is that DSB kinetics are largely unaffected by introduction of the antibody. This predicted behaviour has been validated experimentally using neutral comet experiments (see appendix B). The model therefore supports the hypothesis that the use of anti-*γ*H2AX antibody to quantify DSBs does not violate the image tracer principle.

### 4.2. Auger electron therapy

#### 4.2.1. Problem outline

In addition to *γ* photons that allow SPECT imaging, ^111^In emits short-pathlength, densely ionizing Auger electrons that have the potential to cause complex DNA damage when radionuclide decay occurs in the nucleus [19]. In previous experimental work, it has been demonstrated that when ^111^In-anti-*γ*H2AX-TAT, labelled to high specific activity (i.e. a large amount of ^111^In per unit of antibody), accumulates at DSB sites, it amplifies the DNA damage, decreases clonogenicity, and inhibits tumour growth [17]. In this section, we use the parametrized model defined in §2.2 to investigate this phenomenon.

#### 4.2.2. Model extension and application

To investigate DSB and *γ*H2AX foci dynamics upon introduction of ^111^In-anti-*γ*H2AX-TAT antibody, the model developed in §2.2 is extended to include the formation of *de novo* DSBs as a result of Auger electron irradiation from ^111^In-anti-*γ*H2AX. By considering a population of *N* DNA sites and assuming that each molecule of antibody bound *γ*H2AX initiates new DSBs at rate *k*_9_, we obtain, after following a similar procedure to that outlined in §2.2 (see appendix C):
4.3
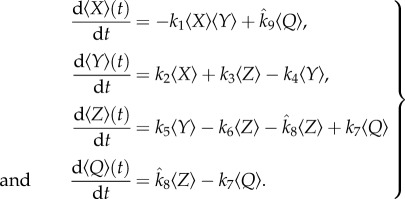
We make the assumption that the probability a given ^111^In-anti-*γ*H2AX-TAT molecule initiates a DSB is proportional to the specific activity of ^111^In, *R*. Hence
4.4
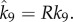


**Figure 8.**
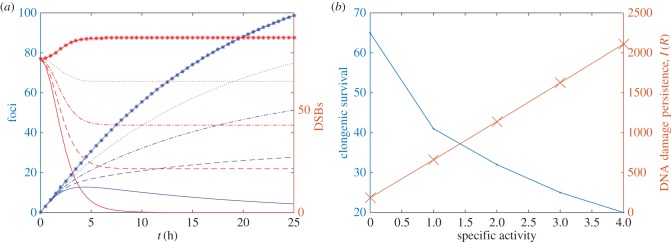
(*a*) Number of DSBs and *γ*H2AX foci are plotted against time at increasing values of parameter 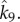 Equations (4.3) and (4.5) were solved for different values of *R*. Solid lines, *R* = 0; dashed line, *R* = 2; dot-dashed line, *R* = 4, dotted line, *R* = 6, asterisked line *R* = 8. (*b*) Experimental measurements of clongenic survival (crosses) and model calculations of AUC (dots) are plotted against specific activity. (Online version in colour.)

Numerical solutions of equations (4.3) for different values of specific activity *R* are presented in figure 8.

#### 4.2.3. Results and interpretation

To compare the model results presented in figure 8*a* with experimental observations, we define the quantity
4.5
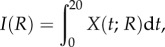
as a measure of the amount and persistence of DSBs. In figure 8*b*, we show that persistence and specific activity are positively correlated.

While DNA damage persistence cannot be measured directly experimentally, we note that cell survival has previously been reported to be inversely correlated with levels of DNA damage (e.g. [20,21]). Furthermore, we have previously measured an inverse correlation between the clonogenic survival of MCF7 cells after exposure to ^111^In-anti-*γ*H2AX-TAT and specific activity of ^111^In (figure 8) (*R*^2^ = 0.97). These observations suggest that the number of DSBs is positively correlated with specific activity, hence providing qualitative support for the model prediction.

## 5. Discussion

During the process of DNA damage repair, numerous molecules in the repair pathway enter an activated state, recognize DNA damage site, initiate repair and disassemble. Via the use of, for example, antibodies that recognize repair processes, the kinetics of repair can be measured. From such data, one can attempt to formulate models of the crucial events that underly the repair process.

Mathematical models allow one to unambiguously formulate and test hypotheses. In the context of the modelling of the DNA repair pathway, there are two well-developed schools. In the first of these, multiple steps in the repair pathway are described. This approach allows one to account for what is known about the numerous molecular players in the system and formulate hypotheses about their mutual interaction. In the latter, the kinetics of foci appearance/disappearance are described but not explicitly the molecular detail.

This study was motivated by a set of experiments in which the introduction of an antibody alters the kinetics of *γ*H2AX foci. To investigate this behaviour, we developed a minimal, stochastic model of essential interactions at the molecular scale. Given that experiments are averaged over thousands of DSBs, we derived ODEs that describe average behaviour within the stochastic model. Using existing experimental data from two cancer cell lines, the parameters in the stochastic model were determined for both cases. We note that the MCF-7 breast cancer cells conform to the repair behaviour observed in most cancer cell lines, where foci appear soon after irradiation, as shown in figure 4*b*. On the other hand, the MDA-MB-468 cells show much delayed repair kinetics, evident from figure 4*a*, and consistent with our earlier data regarding this cell line [12,17,22].

Having developed a model that can explain observations in a non-perturbed case, we extended it to investigate behaviour upon the introduction of anti-*γ*H2AX antibody. A prediction of the model is that the measured rate of formation of antibody-bound *γ*H2AX ought to increase linearly with antibody concentration. This behaviour was found in the experiments at low antibody concentrations. Importantly, the model predicts that the modified foci kinetics are not accompanied by significant changes to the DSB kinetics. This behaviour is also observed experimentally. Hence the model supports the hypothesis that the use of anti-*γ*H2AX antibody to image DNA damage does not violate the image tracer principle. Interestingly, the model indicated the existence of a feedback mechanism, whereby more H2AX is phosphorylated to compensate for *γ*H2AX masked through anti-*γ*H2AX-TAT binding. These effects are consistent with and account for the increased number of foci found after exposure of irradiated cells to anti-*γ*H2AX-TAT.

The presence of ^111^In-labelled anti-*γ*H2AX-TAT can deliver short pathlength, highly ionizing Auger electron irradiation specifically to sites of existing DNA damage, resulting in the induction of new DNA damage. To investigate this phenomenon, we developed the existing model to allow for new DSB induction at a rate proportional to the amount of labelled anti-*γ*H2AX-TAT antibody. By assuming that the induction rate is proportional to the specific activity of the ^111^In, the model can predict the DNA damage load as a function of specific activity. Although this quantity cannot be measured directly in experiments, we used measurements of the clonogenic survival of MCF7 cells as a proxy for DNA damage and found good qualitative agreement between the model and experimental observations.

An assumption made, both in this study and others, while fitting model parameters to experimental counts of *γ*H2AX foci number is that the number of observable foci is proportional to the total number of *γ*H2AX molecules. Using the SSA, we tested this assumption by assuming a *γ*H2AX focus becomes visible under the microscope when the number of *γ*H2AX molecules at a site exceeds a certain threshold. Hence, within the context of the stochastic model, we could count the number of observed foci and the mean number of *γ*H2AX molecules. In our approximation, we found that these quantities did scale with one another, thereby validating this assumption. However, this point raises the issue that in this and similar studies, foci kinetics, which depend on, for example, imaging parameters that determine whether or not a focus is detected, are used to infer details of underlying molecular networks. Measurement of absolute molecule numbers would allow models to be further tested and, for example, the parameters in table 2 to be explicitly measured.

The theoretical framework adopted in this study could be readily extended to account for a more accurate representation of molecular networks regulating DSB repair. For example, instead of assuming there is a single diffusible species that binds to a DSB site, phosphorylates H2AX and is solely responsible for the rate of DNA repair, the variable *Y*(*t*) could represent a vector of *N* molecular species that contribute to the DNA repair rate. Furthermore, our treatment of DNA repair could be modified to account for persistent DSBs that do not appear to undergo repair. We have not addressed these issues in the current work as there are not currently data to constrain the additional parameters.

While modelling the induction of new DSBs as a result of the presence of ^111^In-anti-*γ*H2AX-TAT antibodies, we have used a mean-field assumption in which new sites of DNA damage are independent of the spatial location of current DSBs. In reality, one would expect that new sites of DNA damage are strongly correlated with the spatial location of current sites as Auger electrons decay over short distances. This topic will be explored in a future publication.

## Figures and Tables

**Figure 9.**
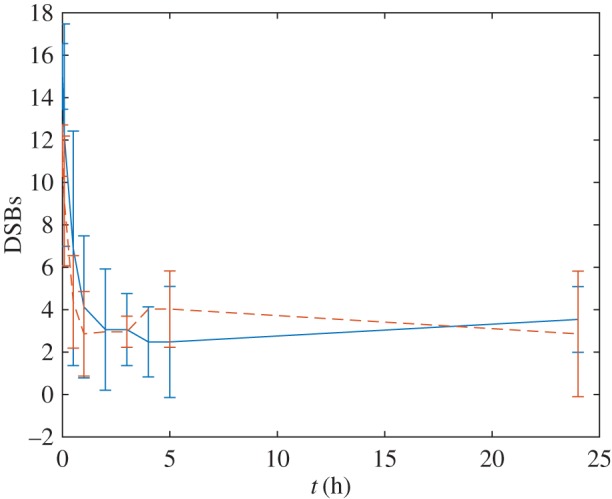
Amount of DNA DSBs, measured using a neutral comet assay, plotted against time post-irradiation for control (solid line) and 0.5 µg ml^−1^ anti-*γ*H2AX-TAT (dashed line). (Online version in colour.)

## Appendix A. Model development for anti-*γ*H2AX antibody

We assume that the probabilities of antibody binding and unbinding with *γ*H2AX in the time interval [*t*, *t* + Δ*t*] are given by

A 1

and

A 2

respectively, where *T*(*t*), *Z*(*t*) and *Q*(*t*) are the numbers of unbound anti-*γ*H2AX-TAT, anti-*γ*H2AX-TAT bound to *γ*H2AX, and *γ*H2AX molecules, respectively, at time *t*; and *k*_7_ and *k*_8_ are rate parameters.

We define *P*(*X*, *Y*, *Z*, *Q*; *t*) to be the probability that at time *t*, a DNA site is in state *X*, with *Y* molecules of bound pATM, *Z* molecules of phosphorylated H2AX and *Q* molecules of antibody-bound phosphorylated H2AX. Given the stochastic processes outlined in the main text, a master equation describing the evolution of *P*(*X*, *Y*, *Z*, *Q*; *t*) is given by

A 3

Following the procedure outlined in §2.2,  and  are defined to be the mean numbers of free antibody and bound antibody–*γ*H2AX complex, respectively. Making the additional assumption that the total amount of antibody is conserved,

A 4

where [TAT]_0_ is the total antibody concentration, and following a similar procedure to that outlined in §2.2, we obtain:

A 5

Making the further additional assumption that free anti-*γ*H2AX-TAT antibody is always in excess of its substrate (*γ*H2AX), i.e.

A 6

equations (A 5) simplify to

A 7

where we define

A 8

## Appendix B. Neutral comet assay following anti-*γ*H2AX-TAT treatment

Using a neutral comet assay as a readout for the relative amount of DNA DSBs, we did not observe a significant difference in the Olive tail moment after irradiation following treatment of MCF-7 cells with or without the addition of 0.5 µg ml^−1^ anti-*γ*H2AX-TAT (*p* = 0.29, figure 9).

## Appendix C. Model development for anti-*γ*H2AX antibody tagged with ^111^In.

To consider the induction of new DSBs, we consider a population of N DNA sites and define *P*(…, *X_i_*, *Y_i_*, *Z_i_*, *Q_i_*,…;*t*) to be the probability that at time *t*, the *i*th DNA site is in state *X_i_*, with *Y_i_* molecules of bound pATM, *Z_i_* molecules of phosphorylated H2AX and *Q_i_* molecules of antibody-bound phosphorylated H2AX. Given the stochastic processes outlined in the previous paragraph, a master equation describing the evolution of *P*(*X_i_*,*Y_i_*,*Z_i_*,*Q_i_*;*t*) is given by

C 1

Assuming that each site has an equal initial probability of being a DSB, the ensemble average at each of the *i* sites will be identical. Hence, dropping the subscripted notation, the governing equations are given by

C 2

Assuming that

C 3
